# Immunogenicity of decellularized extracellular matrix scaffolds: a bottleneck in tissue engineering and regenerative medicine

**DOI:** 10.1186/s40824-023-00348-z

**Published:** 2023-02-09

**Authors:** Mohammadreza Kasravi, Armin Ahmadi, Amirhesam Babajani, Radman Mazloomnejad, Mohammad Reza Hatamnejad, Siavash Shariatzadeh, Soheyl Bahrami, Hassan Niknejad

**Affiliations:** 1 Department of Pharmacology, School of Medicine, Shahid Beheshti University of Medical Sciences, Tehran, 1985711151 Iran; 2 Gastroenterology and Liver Diseases Research Center, Research Institute for Gastroenterology and Liver Diseases, Shahid Beheshti University of Medical Sciences, Tehran, Iran; 3 Department of Surgery, University of California Los Angeles, Los Angeles, California USA; 4 Ludwig Boltzmann Institute for Experimental and Clinical Traumatology in AUVA Research Center, Vienna, Austria

**Keywords:** Decellularization, Tissue engineering, Extracellular matrix, Translational medicine, Immune response, Biologic scaffold, Recellularization

## Abstract

Tissue-engineered decellularized extracellular matrix (ECM) scaffolds hold great potential to address the donor shortage as well as immunologic rejection attributed to cells in conventional tissue/organ transplantation. Decellularization, as the key process in manufacturing ECM scaffolds, removes immunogen cell materials and significantly alleviates the immunogenicity and biocompatibility of derived scaffolds. However, the application of these bioscaffolds still confronts major immunologic challenges. This review discusses the interplay between damage-associated molecular patterns (DAMPs) and antigens as the main inducers of innate and adaptive immunity to aid in manufacturing biocompatible grafts with desirable immunogenicity. It also appraises the impact of various decellularization methodologies (i.e., apoptosis-assisted techniques) on provoking immune responses that participate in rejecting allogenic and xenogeneic decellularized scaffolds. In addition, the key research findings regarding the contribution of ECM alterations, cytotoxicity issues, graft sourcing, and implantation site to the immunogenicity of decellularized tissues/organs are comprehensively considered. Finally, it discusses practical solutions to overcome immunogenicity, including antigen masking by crosslinking, sterilization optimization, and antigen removal techniques such as selective antigen removal and sequential antigen solubilization.

## Introduction

Organ transplantation has remained the last therapeutic choice to treat many end-stage diseases. Based on the recent U.S. Organ Procurement and Transplantation Network (OPTN) report, more than 106,000 people are on the national transplant waiting list, and 17 people die each day due to the lack of suitable organs [1]. Moreover, most transplants have a limited lifetime and finally fail because of immunologic rejections. The complications are mainly attributed to the major histocompatibility complex molecules 1 (MHC I) and 2 (MHC II) (polymorphic antigens expressed on the membrane of all nucleated cells and specialized antigen-presenting cells (APCs), respectively), which trigger a cellular immune response against both allografts and xenografts. MHC I activates CD8^+^ T cells, while MHC II stimulates CD4^+^ T cells [2]. CD8^+^ T cells directly invade immunogenic cellular targets, while recruitment of other inflammatory cells and cytokine production are the major consequences of CD4^+^ T cell activity [3].

On the other hand, the initiation of adaptive immune response and rejection process is not restricted to mismatched MHC molecules. Any heterogeneous antigen that could be processed and expressed through MHC molecules is a potential immunogen [4]. There are various antigens other than MHC molecules, so-called minor histocompatibility antigens, which interact with MHC molecules to activate T cells. Minor antigen-mismatched grafts could be potent enough to cause rejection in MHC-matched grafts [5]. Regarding vast inter/intraspecies genetic polymorphisms, these minor antigens augment the rate of rejection in xeno- or allotransplantation [6, 7].

T cells recognize these extrinsic antigens via the interaction of their receptors with foreign MHC molecules or processed antigen fragments on the MHC molecule clefts [8]. The process of foreign antigen presentation to T cells, called allorecognition, initiates cellular immune response via three pathways: direct, indirect, and semi-direct [9]. The direct pathway provokes the most vigorous immune response by directly representing MHC I and MHC II molecules of the donor’s APCs to recipient CD8 and CD4 T cells, respectively. Activated recipient T cells migrate to the graft site, directly recognize foreign antigens, and participate in acute rejection. This response vanishes within a few weeks because of a limited number of donor APCs and their depletion over time [10]. The indirect pathway of allorecognition is mediated via digestion and processing of donor MHC molecules or minor antigens by recipient APCs, which represent foreign antigens to recipient T cells in the format of foreign peptide-host MHC molecule. Hence, T cells activated through an indirect route have a restricted ability to recognize donor MHC molecules; they only participate in graft destruction through CD4^+^ T cell-mediated cytokine production rather than direct invasion [11, 12]. Although this pathway invokes a less vigorous response, it lasts longer due to the available supply of host APCs and foreign antigens within the grafts. Moreover, CD4 T cells have a substantial role in regulating both humoral and cytotoxic T cell responses as the main elements of chronic rejection. Accordingly, indirect pathway activity is crucial to graft fate [10]. In the third pathway of allorecognition (semi-direct), recipient dendritic cells (DCs) capture intact donor MHC molecules and express them without processing them on their surface. Transfer of donor MHC molecules to the cell surface of recipient DCs occurs during direct cell contact or shuttling of MHC proteins through extracellular vesicles. It is unclear how long this pathway runs, but it does not seem to last for an extended period after transplantation [13–15]. Upon transplantation of viable organs, all the aforementioned pathways of allorecognition are recruited in graft rejection. These significant hurdles associated with immunologic rejection, alongside shortages of supplying allogenic grafts and the risk of possible transmission of pathogens, have increased the demand to find more immune-tolerable alternatives for allotransplantation [16, 17].

In recent years, various synthetic and natural scaffolds have been applied in tissue engineering and regenerative medicine to design tissues and organs repopulating with autologous or stem cells to overcome these immunologic barriers [18–21]. Choosing an appropriate scaffold requires comprehending its interaction with the host immune system. Following the insertion of synthetic scaffolds into the human body, the immune system establishes the foreign body response (FBR), initiated by the migration of neutrophils and macrophages and followed by the production of inflammatory cytokines at the implantation site [22]. Within a few days, attracted macrophages, mostly from inflammatory phenotype (M1 macrophages), form foreign body giant cells, which release potent enzymes and accelerate graft degradation [23]. Consequently, most synthetic grafts are covered with a dense fibrotic capsule and isolated from the human body. FBR remains as long as the graft is fully degraded or removed; otherwise, chronic inflammation may occur [22, 24].

These shortfalls have turned the trend toward applying natural scaffolds, which are tissue/organ decellularization derivatives to produce extracellular matrix (ECM) based scaffolds. Opposite to FBR, natural scaffolds undergo a different positive form of host response, in which ECM experiences lysis and releases some cryptic peptides and growth factors during early degradation. This response phase has a substantial role in shifting macrophage polarization toward reconstructive phenotype (M2 macrophages), angiogenesis, stem/progenitor cell migration, and collectively, the regeneration process. Moreover, some cryptic peptides exhibit antimicrobial traits, resulting in low pathogen-related immunogenicity in decellularized ECM (dECM) scaffolds [25, 26]. Desirable biocompatibility of natural scaffolds is primarily ascribed to the inter- and intra-species conservation of ECM [27] and its MHC-free nature that makes the host immune system recognize them as self-components [28]. Even if small amounts of MHC molecules remain after the decellularization process, the elimination of donor APCs, cell interactions, and presumably extracellular vesicles after the decellularization process would protect dECM scaffolds from the direct and semi-direct pathways and restricts allorecognition to the indirect pathway, which is privileged from acute rejection. Of note, as the final consequence of allorecognition, CD4^+^ T cells are maturated and differentiated into some effector subtypes with distinct functionality [29]. dECM scaffolds affect CD4^+^ T cell maturation and differentiation to shift T cell polarization toward regulatory phenotype (T reg), an immunomodulatory subtype that downregulates T cell activity. Thereby, dECM-based scaffolds are more biocompatible and confront less rejection rate compared to other implants [30–32].

Despite the widespread use of decellularized tissues/organs, there is no sufficient insight into the impact of different steps on their production and modifications regarding the immunogenicity of final products. The application of many dECM scaffolds still encounters immunologic hurdles, hampering their translation into the clinic [33]. This review aims (i) to outline the contribution of the innate and adaptive immune system in the rejection of dECM scaffolds, (ii) to evaluate etiologies of immunogenicity in the dECM scaffolds, and (iii) to provide new insight into different post-processing methods such as antigen removal techniques, crosslinking and sterilization to overcome the immunogenicity challenges in dECM scaffolds.

## Immune mechanisms that affect decellularized tissues/organs

A general understanding of the dECM scaffolds’ interaction with innate and adaptive immunity is a prerequisite for designing tolerable bioscaffolds. The interaction of dECM scaffolds with the innate immune system is mainly mediated via damage-associated molecular patterns (DAMPs), danger signals released upon the cell and ECM damage during graft processing or implantation. Nuclear and mitochondrial DNA, reactive oxygen species (ROS), and fragmented ECM components such as hyaluronic acid or fibronectin are among the most renowned DAMPs, which are released due to perturbation in the arrangement of nucleic acids within cells, transplantation imperfections such as ischemia-reperfusion, and harsh decellularization strategies, respectively [34–37]. DAMPs are recognized by a group of transmembrane and cytoplasmic receptors on various non-immune and innate immune cells, namely, pattern recognition receptors (PRRs) [38]. After recognition by PRRs, DAMPs promote the transcription of genes contributing to the inflammation process. The release of pro-inflammatory cytokines, including interleukin (IL) 1, IL6, and tumor necrosis factor (TNF), as well as chemokines, results in the recruitment of immune cells [39, 40]. The increase in the production of pro-inflammatory cytokines also induces a shift in the polarization of macrophages as the critical determinant of grafts’ long-term prognosis toward the M1 phenotype [41, 42]. Innate immune response provoked by DAMPs accompanies some extent of graft destruction. However, innate immunity is insufficient for graft rejection and immunological traits of the implant, and its interplay with adaptive immunity will determine its fate [34, 39, 43].

**Fig. 1:**
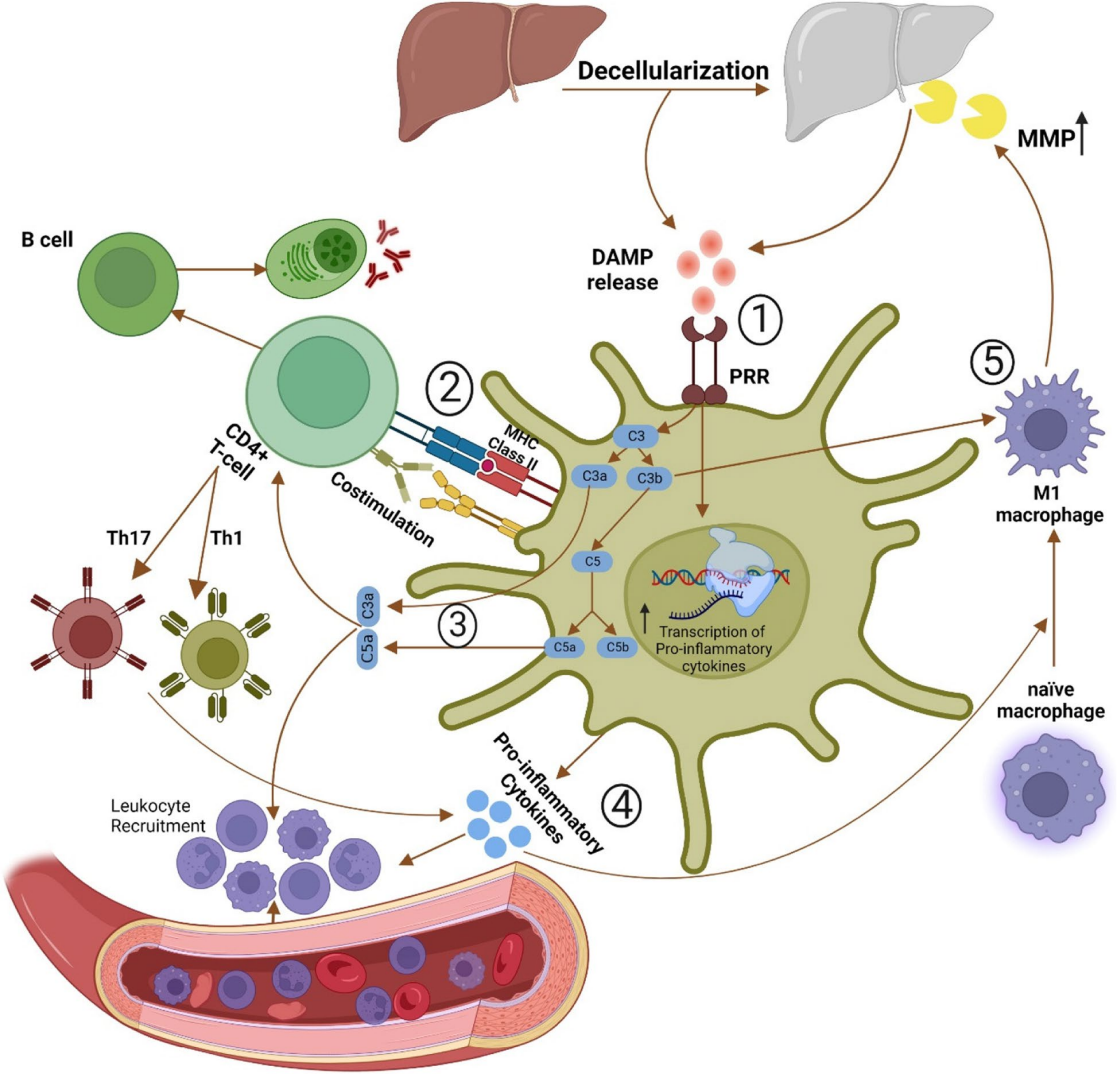
Innate and adaptive immune response against dECM scaffolds. (1) Decellularization strategies inevitably produce various amounts of damage-associated molecular patterns (DAMPs), which trigger pattern recognition receptors (PRRs) on innate immune cells. (2) Stimulation of PRRs in antigen-presenting cells provides prerequisites of T cell activation, including pro-inflammatory cytokine and major histocompatibility complex and co-stimulatory molecules, as well as triggering complement cascade to produce C3a, C3b, and C5a. T cell activation is the essential trigger for B cells, which mediate antibody production against remaining antigens within dECM scaffolds. Antigen-antibody interactions further trigger complement cascades and implement a vicious cycle. (3) C3a and C5a recruit immune cells and induce T helper type 1 (TH1) and TH17 polarization in helper CD4^+^ T cells, increasing the production of pro-inflammatory cytokines. (4) Elevation of pro-inflammatory cytokine content in the implantation site leads to further recruitment of immune cells and induces M1 polarization in naïve macrophages. (5) C3b complements enhance matrix metalloproteinases (MMP) production in M1 macrophages, accelerating the decellularized extracellular matrix (dECM) scaffold degradation and failure

T cells, as the primary mediators of the adaptive immune response, require the simultaneous presence of three signals for their activation, including donor antigens, co-stimulation, and pro-inflammatory cytokines [8]. The decellularization process usually leaves some amounts of antigens, such as MHC molecules and minor histocompatibility antigens, within the derived dECM. Decellularization-induced DAMPs increase the expression of MHC II and co-stimulatory molecules on recipient APCs and facilitate the recognition of these antigens by T cells [39]. Moreover, activation of complement cascades by DAMPs in APCs releases C3a, C3b, and C5a components. C3a and C5a are effective chemoattractants and induce T helper 1 (Th1) and Th17 polarization in CD4^+^ T cells [39, 44]. Th1 and Th17 per se increase the production of pro-inflammatory cytokines and aggravate inflammatory response via induction of M1 polarization in naïve macrophages [45]. C3b components bind their corresponding receptors on these M1 macrophages and trigger the release of matrix metalloproteinases (MMPs), the principal ECM degradative enzyme, mediating the dECM graft failure [46, 47]. Besides, T cell activation is also necessary to mature B cells to produce antibodies against different antigens [45]. Interaction between antigens within dECM transplants and produced antibodies further activates the complement cascade and creates a vicious cycle with the output of dECM scaffold failure [47]. Accordingly, inhibiting excessive DAMP release mainly via preservation of the ECM structure during decellularization, alongside a robust antigen removal process, are the key factors to escape destructive innate and adaptive immune responses (Fig. 1).

## Contributing factors in the immunogenicity of ECM-based biomaterials

Several factors influence the host immune response against dECM bioscaffolds. In this regard, decellularization efficiency and applied method, ECM alteration, tissue origin, implantation site, and recipient features are the main factors determining the host immune response against decellularized tissues [48–50].

### The role of decellularization efficiency in the immunogenicity of dECM scaffolds

Considering the role of foreign cells in graft rejection, decellularization significantly attenuates the host immune response against transplants. Decellularization is defined as removing DNA and other cellular materials while preserving ECM configuration and composition. It is the most effective method to reduce the immunogenic content of tissues and organs. However, incomplete removal of cell components during the decellularization process may provoke severe post-implantation immune responses [51, 52]. To define a competent decellularization process, two criteria were introduced in which dECM should neither have visible nuclear contents under hematoxylin and eosin (H&E) and 4′,6-diamidino-2-phenylindole (DAPI) staining, nor exceed 50 ng and 200 base pairs threshold for remaining dsDNA content (per dry weight) and length, respectively [53]. The quantification of residual DNA as a potent immunogen and indirect index of remaining cell materials is a practical tool to estimate the remaining immunogen amount throughout the dECM [54–56].

However, the residual DNA within dECM cannot elicit rejection in the absence of immunogenic adjuvants [57], and US Federal Drug Administration allows the unrestricted presence of DNA in biological scaffolds [58]. The application of some commercially available biomaterials, which contain more than 50 ng/mg of remaining DNA, has shown good tolerance [48]. Besides, Böer et al. showed that endonuclease treatment, as a popular agent applied in many decellularization protocols, could misunderstand us and cause DNA elimination while other cellular components still exist in the scaffold. Although their decellularization protocol virtually eliminated DNA and significantly reduced cellular proteins, they quantified over 300 cellular proteins, including ingredients of cytosol, cell membrane, organelles, nucleus, and cytoskeleton. These remaining proteins provoked a high immunogenic response in their in vivo study [54]. Retention of some of these cellular proteins, such as vimentin, which has inherent antigenicity, may contribute to decellularized tissues’ immunogenicity [59]. On the flip side, the complete removal of cellular materials necessitates the application of robust decellularization, which may accompany unwanted consequences. Such protocols damage ECM ultrastructure and deplete favorable entities that consequently have detrimental effects on yielded scaffold function and regenerative properties [51, 52]. For instance, laminin, collagen IV, and fibronectin loss may impair angiogenesis [60, 61], and glycosaminoglycans (GAGs) removal accompanies growth factors depletion, impairing subsequent cell migration and differentiation [53]. Additionally, ECM alterations may turn it into a source of immunogenicity in decellularized tissues [59]. Taken together, there is no consensus for accepting the abovementioned criteria as a standard to predict decellularized scaffolds’ immunogenicity. The lack of a comprehensive measure to evaluate the functionality of ECM-derived bioscaffolds made it quite arbitrary whether a biomaterial is well suited for clinical translation or not [62].

### The role of decellularization methods in the immunogenicity of dECM scaffolds

Apart from the decellularization process’s inefficacies in removing cellular materials, its detrimental effects on the immunogenicity of derived scaffolds are mediated via two primary mechanisms. First, as discussed below, perturbations in the ECM integrity may directly increase its immunogenicity [63]. The second mechanism is related to the ECM alterations, which impede subsequent recellularization [51] and consequently increase the availability of present antigenic sites for the host immune system [64]. Due to the critical role of subsequent recellularization in decreasing ECM immunogenicity, certain issues should be considered in choosing an appropriate protocol among various physical techniques and chemical and biological agents, including cytotoxicity of agents, applied washing methods, and depletion of growth factors during decellularization [65]. Herein, the contribution of common decellularization strategies to the immunogenicity of derived scaffolds is discussed.

#### Physical decellularization

Physical decellularization techniques employ temperature, pressure, and other physical characteristics to lyse cell membranes and facilitate cell removal. Notably, they are usually incompetent to decellularize tissues/organs thoroughly and leave significant amounts of immunogen cell remnants [66]. Nonetheless, their less toxicity makes them attractive options for adjuvant treatment for enhancing the chemical and biological agent effectiveness [67]. Among physical decellularization techniques, immersion and agitation, and perfusion are of the highest importance as widely utilized routes for delivering chemical and biological agents. Immersion and agitation of tissues in chemical and biological agents is a relatively simple decellularization method. It uses physical stresses to enhance agent effects on intended tissues. However, its efficacy is relatively low for removing the immunogenic cellular content of large organs or dense tissues [68]. On the other hand, perfusion exploits a pump to induce an artificial circulation of chemical and biological agents through the whole organ vasculature. It is an appropriate route to decellularize larger organs. Perfusion efficacy in removing immunogenic cellular remnants and its effect on ECM correlates with infusion direction (antegrade or retrograde), selection of route (artery or vein), physical parameters such as flowrate, pressure, and temperature, and properties of the applied agents [69]. Herein, the contribution of various physical decellularization techniques to the immunogenicity of ECM-based biomaterials is summarized in Table 1.

**Table1:** Common physical decellularization methods and their influence on the immunogenicity of derived bioscaffolds

Method	Advantages	drawbacks	Ref.
**Freeze and thaw cycles**	↓ DAMP release via reducing detergent treatment time ↑ cell removal in tissues with dense mechanical barriers (i.e., osteochondral tissue)	Inefficient antigen removal	[56] [70] [71]
**Non-thermal electroporation**	↑ cell removal ↓ ECM damage and DAMP release	Cytotoxicity of some applied solvents	[67, 69]
**High hydrostatic pressure**	↑ cell membrane lysis at high pressures (above 150 MPa) ↓ pathogen-related immunogenicity via simultaneous sterilization at 900 MPa	Protein denaturation at pressures higher than 600 MPa Compromising the dECM mechanical properties	[72] [73] [74]
**Mechanical sonication**	Exploiting shear stress effect to lyse cell membrane ↑ efficacy of chemical and biologic agents	Disruption in ECM structural fibers ↑ exposing antigenic sites	[69, 72]
**Mechanical agitation**	↑ removal of immunogenic cell debris	Ineffective for removing immunogenic cell materials from large organs and dense tissues	[72] [75]
**Perfusion**	↑ delivery of chemical and biologic agents ↑ removal of antigens and immunogen cell debris	Only applicable in organs with innate vasculature Disrupting ECM at high flow rates	[76, 77]
**Supercritical CO2**	Non-cytotoxic nature Quick decellularization time Well preservation of ECM ↓ pathogen-related immunogenicity via simultaneous sterilization	ECM denaturation due to use of co-solvents	[56] [78]
**Vacuum assistance**	↑ DNA and α-gal epitope removal ↓ detergent treatment time ↓ ECM denaturation and DAMP release ↑ scaffold porosity and recellularization process	Insufficiency and need for chemical and enzymatic co-treatment	[79, 80]

#### Chemical and biological agents

Chemical and biological agents are key players in the decellularization process. These agents exert distinctive influences on the biocompatibility and immunogenicity of yielded scaffolds. Hence, understanding the impact of each agent on derived ECM scaffolds aids us in elaborating decellularization techniques to minimize the adverse effects of acids and bases, non-ionic, ionic, and zwitterionic detergents, besides chelators and enzymatic agents as the most widely utilized agents, on the immunogenicity of dECM scaffolds.

##### Acids and bases

Peracetic acid (PAA) and sodium hydroxide (NaOH) are the prominent representatives of acids and bases, respectively. PAA’s adverse effects on ECM structure and composition and later repopulation seem negligible; however, it is not a strong agent in removing immunogen cell materials and is mainly used for sterilization [48, 55]. Comparatively, NaOH, as a typical alkaline, extracts cellular components efficiently. It appears to be a cytocompatible agent for subsequent recellularization, but it significantly damages ECM structure and decreases growth factors’ content. Alkalines such as NaOH separate DNA strands at pH>11 and extract cell components, but decellularization at higher pH provokes a more robust immune response than in neutral conditions. Presumably, higher pH shatters ECM and produces particles that elicit more severe immune reactions [81, 82]. Notably, more aggressive protocols designed for decellularization in higher pH diminish fibronectin, laminin, elastin, and GAGs’ content and integrity, which in turn interfere with subsequent cell adhesion and proliferation [82]. Accordingly, pH in ranges far from the neutral zone better removes cellular components, whereas its deleterious impacts on the ECM will enhance the immunogenicity of derived scaffolds. To optimize pH in decellularization protocols, the organ-specific structure and expected functionality of each scaffold should be considered.

##### Non-ionic detergents

Non-ionic detergents such as Triton X 100 (TX100) disrupt lipid-lipid, lipid-protein, and DNA-protein bonds while preserving protein-protein interactions. They disintegrate membrane proteins and lyse cells without altering the ECM protein structures. TX100 effects are mainly dependent on its concentration. Higher TX100 concentrations will cause unfavorable effects on ECM [51, 83]. TX100 remove GAGs and decreases laminin and fibronectin content, but it does not affect collagens, showing its detrimental effects on ECM are milder than their ionic counterparts [75, 84]. Moreover, considerable research indicates TX100 is cytotoxic, necessitating TX100-derived ECM safety control before clinical translation [85]. Retention of TX100 within dECM should be minimized by applying lower concentrations and different washing steps to achieve scaffolds with less than 15mM remaining TX100 concentrations, which was previously shown to get tolerated well by human cell lines [86].

It is of interest to further note that TX100 decellularization efficacy is highly dependent on the target tissue configuration. Complete decellularization has been reported in thin tissues such as nerves and pericardium, while TX100 application alone does not seem successful in removing immunogen cell debris from denser tissues such as arteries [56, 75, 83]. Moreover, TX100 breaks DNA down into larger fragments than ionic detergents, which is disadvantageous in terms of the immunogenicity of dECM scaffolds [55].

##### Ionic detergents

Ionic detergents such as sodium dodecyl sulfate (SDS) and sodium deoxycholate (SDC) target protein-protein bonds and efficiently detach cellular proteins and nuclear materials. However, they may alter ECM architecture and disrupt the structure of protein constituents, including collagens [51]. GAGs and growth factor depletion are major issues that should be considered when using ionic detergents due to their impact on compromising future recellularization [87]. This ECM denaturation may alter the composition, mechanical features, and morphology to impede regenerative processes and provoke adverse immune reactions and FBR [88].

SDS is the most powerful detergent for removing cytoplasmic proteins and antigens [48]. However, retention of SDS within decellularized tissues promotes inflammation and FBR following implantation. This reaction is partially explained by the cytotoxic effects of remaining SDS for the recellularization and regeneration process [88, 89]. Accordingly, SDS needs to be efficiently washed out of treated scaffolds. SDS concentrations over 50 mg/L in washing solution or remaining SDS in ECM-based biomaterials beyond 10 µg/mg dry weight have shown cytotoxicity [56, 87]. However, strong hydrophobic bounds between SDS and proteins hamper the elimination of SDS via conventional washing methods. Terminal CaCl2 co-treatment could be exploited to detoxify the remaining SDS via precipitation [89]. TX100 could also be applied to eliminate residual SDS from decellularized tissues, making TX100/SDS a good combination for better cell removal and minimization of cytotoxicity synchronously [55]. Furthermore, the exploitation of negative pressure in vacuum-assisted methods has recently demonstrated efficacy in enhancing SDS removal following the washing step of decellularization [90].

SDC, another ionic detergent, is a potent agent for extracting cellular proteins from dense tissues [55]. Zhong’s study on rabbit trachea endorsed SDC potency in cell removal and ECM preservation in dense tissues, which resulted in lower immunogenicity of derived bioscaffolds compared to TX100-treated peers [83]. SDC is also more biocompatible compared to SDS [48, 91]. However, whether it is less destructive than SDS to ECM is a place of controversy [92].

##### Zwitterionic detergents

Zwitterionic detergents such as sulfobetaine and CHAPS exhibit the properties of a hybrid of ionic and non-ionic detergents. The efficacy of CHAPS in eliminating cell remnants is controversial. Despite promising results in a few organs, such as lungs, accumulated evidence indicates their failure to preserve ECM and eliminate cellular materials in other organs. Of note, they have been demonstrated to deplete the elastin and GAGs content of ECM [51, 75, 82]. Nevertheless, relying on the dual structure of zwitterionic detergents, sulfobetaines have been successfully exploited in solubilization, the process of removing hydrophobic and hydrophilic immunogenic content of tissues/organs [88].

##### Chelating agents and enzymes

Divalent cations such as Ca^2+^ have a fundamental role in the cell-ECM attachment. Chelators such as ethylenediaminetetraacetic acid (EDTA) detach these cations from cell membrane binding sites and release cell components out of ECM [48, 93]. Furthermore, chelators may facilitate removing nuclear immunogens via depleting free divalent cations, which precipitate DNA on ECM [94]. However, mere EDTA treatment is insufficient to achieve suitable acellular ECM scaffolds [95].

Enzymes such as proteases and nucleases are used to remove inter- and intracellular chains and omit unwanted cell elements and their interactions with ECM. However, they would induce an immune response and hamper repopulation if they do not get washed out before application properly [69]. Trypsin is the prevailing protease that is usually used in combination with EDTA. It splits cell-ECM peptide bonds with optimum performance at 37°C and pH=8 [75]. Although the application of trypsin in the initial steps as an adjuvant could enhance decellularization efficacy, the sole application of trypsin is insufficient to eliminate cells. It needs to be utilized with other agents [69, 96]. Trypsin/EDTA, as the most frequently used combination, alters collagen composition and decreases GAGs, fibronectin, laminin, and elastin. Accordingly, optimizing concentration and treatment time is necessary to prevent its destructive effects on ECM [84, 97].

DNases, another used enzyme, efficiently break DNAs into smaller fragments to facilitate their removal. However, difficulties with DNase removal from treated tissues provoke some immunologic concerns and hamper subsequent recellularization attempts [48, 72, 74]. DNase may also exert detrimental influences on ECM composition, as prolongation of DNase treatment time is indicated to deplete major basal lamina constituents, laminin, and collagen IV [98]. Despite DNA, RNA has a short lifetime, and its retention within ECM-based biomaterials does not presumably associate with major immunologic issues [52]. Viral contamination is the substantial cause of ribonuclease treatment in some decellularization protocols [99]. An overview of the impact of chemical and biological agents on the immunogenicity of the natural acellular scaffolds is summarized in Table 2.

**Table 2:** Common chemical and biological agents used for tissue/organ decellularization and their immunological impact on derived scaffolds

Method	Typical decellularization agents	Advantages	Disadvantages	Ref.
**Acids**	PAA	Favorable ECM preservation ↓ pathogen-related immunogenicity due to simultaneous sterilization	Weak antigen removal	[48, 55]
**Bases**	NAOH	High potency in removing DNA and other immunogens Cytocompatibility	↑ ECM alteration and DAMP release ↓ growth factor	[81, 82]
**Non-ionic detergents**	TX100	↑ removal of DNA and membrane immunogens	↑ ECM alteration and DAMP release Low efficiency in dense tissues Cytotoxicity	[51, 56, 75, 83–85]
**Ionic detergents**	SDS SDC	High potency in removing protein antigens, especially in dense tissues	↑ ECM alteration and DAMP release Exposing hidden antigenic sites ↓ GAG and growth factor Cytotoxicity Necessitating robust washing methods	[48, 55, 56, 87–89]
**Zwitterionic detergents**	CHAPS Sulfobetaines	Effective agents for solubilizing both hydrophobic and hydrophilic immunogens	↓ GAG and elastin ↑ DAMP release Exposing hidden antigenic sites	[51, 75, 82, 88]
**Chelating agents**	EDTA	↑ cell-ECM dissociation ↑ nuclear material removal	Weak cell and antigen removal efficacy	[48, 93–95]
**Enzymes**	Proteases Nucleases	↑ cell-ECM dissociation Eliminating nuclear antigens	Disrupting the structure of collagen, laminin, GAG, and elastin ↑ DAMP release Exposing hidden antigenic sites ↓ recellularization due to retention of enzymes within dECM	[69, 72, 84, 97, 98]

### The role of ECM alteration in the immunogenicity of decellularized scaffolds

ECM is a reservoir for the cryptic particles that activate or suppress inflammation and orchestrates innate and adaptive immune responses [100, 101]. ECM denaturation during decellularization may enhance the immunogenicity of dECM scaffolds via (i) exposing hidden immunogenic domains to induce immune reactions, (ii) converting inert molecules to immunogen particles, and (iii) depleting immunomodulatory moieties [63].

Denaturation of ECM components via harsh decellularization methods may expose some cryptic helical and terminal antigenic sites of collagen, which trigger antibody production against ECM-based bioscaffolds [48]. Besides, some cryptic laminin motives induce macrophage and neutrophil chemotaxis and upregulate MMPs activity [102]. MMPs per se degrade collagens and expose cryptic three-peptide motives PGP (Pro-Gly-Pro), which trigger CXCR2 receptors of neutrophils and orchestrate their chemotaxis and interplay with adaptive immunity. Unrestricted release of PGP motives in the transplantation of organs such as heart and lung mediate chronic inflammation and rejection [102, 103].

Alteration of inert molecular structures may also influence the immune reaction against transplanted grafts. In normal conditions, elastin, which constitutes approximately two-thirds of elastic ligaments, half of the blood vessels, one-third of the lungs, and 3% of dermis dry weight [104], does not usually undergo turnover [105]. During the decellularization of such tissues, elastin may be denatured that leads to the release of elastin particles; directly via the impact of harsh agents such as SDS and trypsin or indirectly via the depletion of GAGs, which have a critical role in protecting elastin from protease activity [46, 106–108]. The release of elastin particles exposes latent immunogenic determinants such as GXXPG and XGXPG (X could be any hydrophobic amino acid), which are indicated to enhance monocyte chemotaxis, T helper cell differentiation to inflammatory phenotypes (TH1 and TH17) and antibody-mediated inflammatory response [109]. Moreover, glycine-rich sites on elastin particles are calcification nidi, making elastin-containing biomaterials susceptible to calcification upon implantation [110].

In another example, high molecular weight hyaluronic acids (HMWHAs), well-known GAGs of dECM, exert anti-inflammatory effects by impeding antigen-antibody interaction and triggering toll-like receptors (TLRs), a subgroup of PRRs, and CD44 receptors of innate and adaptive immune cells. Activation of these receptors accompanies a decrease in DC maturation and promotion in the polarization of macrophages toward M2 phenotype besides differentiation of T cells into regulatory phenotype and induction of apoptosis in activated T cells. In contrast to HMWHAs, low molecular weight hyaluronic acids (LMWHAs) are released during ECM damage or degradation, which act as DAMPs and drive inflammatory responses such as enhancing immune cells chemotaxis, DCs maturation, and M1 polarization of macrophages via the same receptors (CD44 and TLRs) [63, 100]. Accumulation of LMWHA fragments within lung transplants has been shown to contribute to chronic inflammation and graft rejection [111]. Likewise, ECM contains other immunogenic GAGs, such as versican and biglycan, which stay latent in intact ECM. Upon ECM damage or elevated activity of the proteases, they are released and stimulate TLRs to activate inflammatory responses mainly via the NF-кB pathway, enhancing M1 polarization of macrophages and adaptive immunity activation [112]. Despite data suggesting the release of fragmented ECM components such as elastin and HA as DAMPs upon decellularization [113], the question of how the decellularization process affects the HA and other GAG structures and whether it changes the HMWHA/LMWHA ratio is remained to be elucidated and is a place for future investigations.

**Fig. 2:**
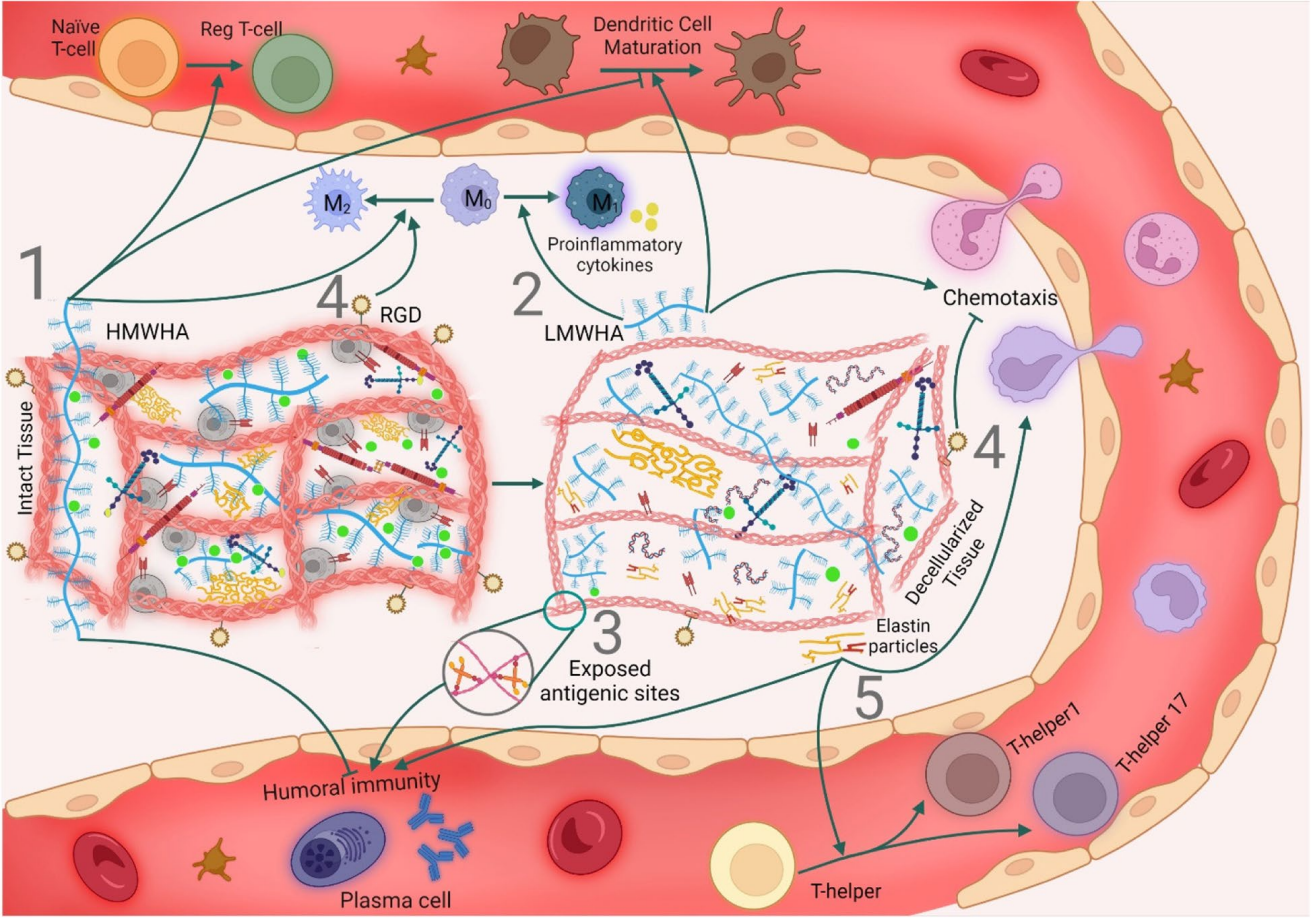
ECM alterations and immune response. Decellularization-derived damages to the extracellular matrix affect its immunogenicity via exposing/producing immunogenic particles or depleting immunomodulatory molecules. (1) Decellularization may destroy immunosuppressive traits of high molecular weight hyaluronic acids (HMWHAs) by breaking them into low molecular weight hyaluronic acids (LMWHAs). HMWHAs induce a regulatory phenotype in T cells and macrophages while suppressing humoral immunity and dendritic cell maturation. (2) LMWHAs are demonstrated to interact with CD44 and toll-like receptors of leukocytes and invoke inflammatory responses such as enhancing leukocyte chemotaxis, dendritic cell maturation and polarization of naïve macrophages toward M1 phenotype. (3) Collagen denaturation during decellularization may expose some cryptic antigenic sites and trigger antibody production, or (4) upregulate immune system activity through the depletion of RGD motives, which impede leucocytes chemotaxis and take part in the M2 polarization of macrophages. (5) Elastin particles are released upon elastin damage and provoke monocyte chemotaxis, antibody-mediated immune response, and T helper cell differentiation toward inflammatory phenotypes

Undesirable effects of decellularization on the immunogenicity of the derived bioscaffolds are not confined to exposing antigenic sites or generating immunogenic DAMPs. Immunomodulatory traits of dECM scaffolds are largely attributed to the ability of their collagens to trigger leukocyte-associated immunoglobulin-like receptors-1 (LAIR-1) of immune cells and consequently downregulation of immune cell activity [100]. Moreover, three-peptide sequence amino acid (Arg-Gly-Asp) domains (RGD motives) of collagen, laminin, and fibronectin interact with integrin receptors of immune cells and exert significant immunomodulatory effects such as dampening adaptive immunity activities, neutrophils chemotaxis and pro-inflammatory cytokine production in macrophages [63]. The ability of ECM peptides to trigger corresponding receptors on the immune cells also depends on the tertiary conformation of molecules [114]. Thus, loss of the immunosuppressor ECM components or perturbation in their 3D structure during the decellularization process may enhance the immunogenicity of ECM-based biomaterials (Fig. 2 and Table 3).

**Table 3:** Contribution of ECM alterations to the immunogenicity of decellularized tissues/organs

**ECM component**	**ECM alteration effect on the immune system**	Ref.
**Collagen**	• Triggering humoral immune response via exposing hidden antigenic sites • Exposing DAMPs (i.e., PGP motives) • Changing the structure or depleting immunosuppressive RGD motives and LAIR-1 ligands	[48, 63, 100, 102, 103]
**Elastin**	• Triggering humoral immune response via exposing hidden antigenic sites • Exposing DAMPs (i.e., GXXPG and XGXPG domains)	[109, 113, 115]
**Fibronectin**	• Changing the structure or depleting immunosuppressive RGD motives	[63]
**Laminin**	• Changing the structure or depleting immunosuppressive RGD motives • Exposing DAMPs	[63, 102]
**GAGs**	• Changing the structure or depleting immunosuppressive high molecular weight hyaluronic acids • Producing highly immunogenic low molecular weight hyaluronic acids	[63, 100, 113]

### The role of tissue/organ sources in the immunogenicity of dECM scaffolds

The source of tissues or organs used to fabricate dECM scaffolds is of the highest importance. Most homologous proteins of different species vary in some amino acid sequences [116]. Human tissues/organs have the least immunogenicity among various bioengineering sources, making allotransplantation the gold standard of treatment for some diseases. However, human sources are not widely available [49]. Therefore, research on the immunogenicity of animal-derived dECM scaffolds has gained much interest.

Interspecies variations between ECM components such as collagen, laminin, fibronectin, elastin, and growth factors are not extreme, making xenogeneic ECM plausible surrogates for their human-derived counterparts [114, 117–119]. Despite the interest in using non-human tissues for transplantations, there are still serious concerns about antigenic glycans, including galactose-alpha-1,3-galactose (α-gal), N-glycolylneuraminic acid (Neu5Gc), and Sda, as the paramount immunogenic sites of xenografts. All mammals except old-world monkeys, apes, and humans express α-gal epitopes on their proteoglycans, glycoproteins, and glycolipids. These epitopes are products of the alpha-1,3 galactosyltransferase enzyme [116], which has been suppressed in the common lineage of these primates. Human intestinal bacteria represent these oligosaccharide moieties on their surface that stimulate the continuous production of antibodies against α-gal epitopes, which account for 1% of serum antibodies [120, 121]. The vast amounts of α-gal in cellular materials are the most stringent barrier in xenotransplantation. Decellularization is a potent means to reduce α-gal-related immunogenicity. Besides eliminating cellular α-gal epitopes, many decellularization methods also reduce ECM-associated α-gal epitopes. However, there are contradictory data about the degenerative effects of remaining α-gal epitopes within decellularized tissues [53, 122, 123]. Despite containing α-gal epitopes, porcine small intestinal submucosa (SIS) ECM has been successfully utilized in the clinic for many years without major adverse immunological events [24, 72]. Nonetheless, α-gal epitopes are identified as one of the main barriers to the clinical application of processed animal bone grafts [124] and tendons [116]. Likewise, several studies have suggested α-gal epitopes as the source of chronic rejection and calcification in bioprosthetic heart valves [125–127]. Xenogeneic porcine heart valve implants failed in a clinical trial due to incomplete decellularization. In this study, four children were treated with Synergraft ™ decellularized porcine heart valve. Unfortunately, three out of four children died from severe inflammatory responses against remaining cell materials, including DNA and α-gal epitopes [128]. To find out about the etiologies of this failure, another study was conducted by Bastian and colleagues. They incubated decellularized porcine heart valves with human plasma in vitro. IgG deposition led to classic complement cascade activation, resulting in polymorphonuclear neutrophil chemotaxis and activation, demonstrating the presence of xenoantigens [129]. Subsequent studies also suggested the retention of α-gal epitopes as an etiology for Synergraft ™ failure [130]. These contradictory results may be explained by the variance of α-gal expression in different tissues and organs [125]. Since porcine decellularized SIS mainly consists of collagen fibers, it does not have many carbohydrate chains and, consequently, α-gals. The existing amounts of these epitopes are presumably insufficient for activating the complement cascade. Therefore, they do not alter regeneration outcomes [116]. Conversely, ECM of tissues such as heart valves, bone, tendon, and cartilage contain abundant amounts of proteoglycans (and consequently α-gal epitopes), which may remain even after a vigorous decellularization process and cause graft failure [47, 116].

Despite undergoing attenuated immune responses, grafts taken from non-human primates or α-gal-free pigs still succumb to delayed antibody-mediated rejection, suggesting the presence of antigens other than α-gals within xenogeneic tissues [131, 132]. Xenogeneic sialic acids are one of the leading non-α-gal antigens, participating in the rejection of xenografts [133–135]. Sialic acids are sugar moieties capping carbohydrate chains in glycolipids and glycoproteins. Neu5Gc and Neu5Ac are the predominant sialic acids expressed in mammals [127, 132]. Deleterious mutations during evolution inactivated the CMAH gene in human and new world monkeys and made their cells exclusively express the acetylated form of sialic acid, Neu5Ac, and produce antibodies against the glycolyl form, Neu5GC [136, 137]. Anti-Neu5Gc antibodies are produced in response to dietary Neu5Gc, non-typeable Haemophilus influenza, or prior animal-derived biotherapeutics exposure and constitute 0.1% to 0.2% of total human antibodies [138]. Although a large body of data indicates the critical role of Neu5Gc antigen-antibody interactions in mediating chronic inflammatory conditions such as cancer and atherosclerosis [127, 138–141], Neu5Gc antigen-antibody interaction is less destructive than the same for α-gals [137]. Data related to non-human primate kidney transplantation into human recipients indicate its privilege from hyperacute rejection and preservation of functionality for at least ten days [131]. The findings could be ascribed to a lower presence of preformed antibodies and IgM/IgG titer alongside inferiority in expressing these epitopes and their affinity to antibodies [137]. Neu5Gc expression level is a tissue-dependent issue. Despite the detrimental effects of these epitopes on the immunogenicity of decellularized heart valves [137, 138, 142], and dermal grafts, neurons are privileged of Neu5Gc expression [131, 137, 143].

Sda antigen, another subtype of glycan antigens, is a known human blood group. Although 95% of people are Sda positive, different structure of pig Sda antigens compared to human and non-human primates has made them xenoantigens, contributing to the xenograft antibody-mediated rejection. Tissues from genetically modified pigs that do not express Sda antigens provoke fewer immune responses in non-human primates and humans [144]. Incubation of pig peripheral blood mononuclear cells with human and monkey sera demonstrated that the Sda antigen is immunogen in a dose-dependent manner. Consequently, revealing the relationship between animal Sda antigen retention within dECM and host immune response would be of importance [145].

Since fibronectin, laminin, and hyaluronic acid as ECM glycoproteins express glycan xenoantigens such as α-gal and Neu5Gc and Sda [72, 146], regardless of decellularization efficacy, xenogeneic dECM scaffolds contain these epitopes which trigger B cells to produce anti-gal antibodies following their implantation and hamper regeneration process [24]. Anti-gal and anti-non-gal antibodies mask the ECM cues substantial for progenitor/stem cell recruitment and their differentiation toward the functional tissues. Moreover, ECM-antibodies interaction enhances macrophage activity and accelerates the degradation of dECM scaffolds, preventing cryptic peptides from releasing at a time sufficient for a good remodeling process [47]. Pre-implantation recellularization of xenografts with human stem cells seems a practical means to attenuate the host immune response against glycan antigens [147].

Additional to immunologic concerns related to glycan antigens, xenogeneic dECM could induce the immune response due to some of their protein constituents. In a study on the immunogenicity of bovine bioprosthetic heart valves, 19 protein antigens, including one ECM protein (Hyaluronan and proteoglycan link protein 3), provoked human antibody response [148]. Moreover, collagen structure comprises triple helix and non-helical regions named telopeptides. Despite the high conservation of triple helical regions in mammalian species during evolution, telopeptides vary significantly interspecies, suggesting them as the main antigenic sites of collagen [114, 149, 150]. In some people, animal collagen (mainly bovine collagen) may provoke immune responses [151]. These inflammatory responses are insignificant and usually subside within a year [114]. Some researchers have proposed enzymatic treatment with pepsin would precisely remove telopeptides without altering triple-helical structures. However, it is arguable whether its application diminishes the immunogenicity of derived biomaterials [114, 152]. Consistently, to fabricate suitable xenogeneic dECM, it is noteworthy to choose animals with the most concordant protein structures [33]. Despite being similar to humans, non-human primates such as chimpanzees are endangered animals. Thus, because of ethical issues [125] and similarities in size, genealogy, and ECM structure, pigs could be appropriate surrogates for human donors. In addition, porcine scaffolds facilitate human cell attachment and their subsequent growth better than other animal-derived scaffolds [153, 154]. However, the porcine endogenous retroviruses (PERVs) gene is integrated within the porcine genome that may be transmitted through resident porcine cells. Therefore, their potential immunogenicity and pathogenesis in humans must be addressed prior to clinical translation [155].

The age of harvested tissue to fabricate ECM-based biomaterials is another source-related determiner of immunologic traits [63]. ECMs derived from aged individuals have less ability to switch the polarization of macrophages from M1 to M2. Consequently, they are associated with chronic inflammation and have less regenerative capacities to support constructive remodeling [122, 156]. The ECM, with aging, will change in structure, mechanical features, and composition. Collagen, GAG, laminin, fibronectin, and growth factor content of dECM age- dependently vary [157]. An example of these changes is the reduction in size and the molecular weight of HA with age. The age-dependent changes usually occur due to elevation in the activity of some proteases, affecting the immunogenicity of derived dECM scaffolds via the abovementioned mechanisms [158].

Tissue-specific degradation rate is the other source-related factor affecting the immunogenicity of dECM scaffolds. Due to the unique properties of each tissue (i.e., specific tissue density), dECM scaffolds from different sources undergo distinctive degradation rates [159]. In this respect, it takes a median of six months for decellularized vessels to undergo ECM regeneration and be fully replaced with new host ECM [25], while SIS-ECM in endarterectomy requires only three months to get fully degraded and replaced with host functional tissues [160]. Therefore, the dECM degradation rate determines the time needed for ECM to be fully replaced with a newly synthesized host ECM. If the graft survives within this period, it can presumably get over the rejection [25]. For this reason, decellularized scaffolds with slower turnover need to withstand longer against immune response; thus, they probably require more robust antigen removal protocols [130].

### The role of the implantation site and recipient features in the immunogenicity of dECM scaffolds

Implantation site and recipient features are the other key factors determining the immune response toward decellularized tissues. The implantation site significantly impacts the host immune response toward dECM scaffolds. Upon implantation of dECM scaffolds, highly vascularized environments such as omentum or sites with direct exposure to the immune system via the bloodstream provoke a more robust immune response toward remaining antigens than less vascularized subcutaneous areas [2, 161]. Accordingly, the absence of blood and lymphatic circulation, the ocular blood barrier, and the presence of immune regulatory factors in the anterior eye chamber result in a relatively lower rejection rate upon cornea transplantation. The need for a longer time for graft immunogens to be recognized by the host immune system, as well as low expression of MHC I and lack of MHC II expression by corneal cells, has made corneal xenotransplantation a far-reaching surrogate for allotransplantation [162].

Since porcine SIS has a long history in human applications, it could be served as an excellent sample to evaluate the impact of the implantation site on the host immune response toward dECM scaffolds. Exploiting porcine SIS as a natural mesh for abdominal wall and inguinal hernia reconstruction has shown promising results over five-year studies [163]. It has also been utilized in other sites with acceptable outcomes [164, 165]. Nonetheless, some studies reported that porcine SIS application for vaginal wall or rotator cuff tendon reconstruction might accompany undesired immune reactions toward graft [166, 167]. Although the underlying mechanisms are not yet fully understood, there is evidence to support the role of T reg cells in the disparities regarding graft acceptance. T reg education in different tissues/organs occurs at different rates. [168]. In a physiological state, T regs account for approximately 30% of total CD4^+^ T cells in the skin and colon, while this proportion is around 10% in skeletal muscles and small intestines [169]. This phenomenon may partially explain the grafts’ unique fate in different implantation sites.

The impact of recipient features such as age and gender on its immune response toward implanted acellular scaffolds seems to be greater than previously thought. In a recent study, Ebken et al. demonstrated that younger male individuals provoke the most vigorous antibody-mediated immune response toward decellularized homograft heart valves, suggesting the critical role of the recipient features in interpreting immunological data [50].

## Solutions to alleviate dECM scaffold immunogenicity

The release of DAMPs during tissue processing and retention of some immunogenic epitopes within ECM, even after complete decellularization, alongside contamination with pathogens, have prompted us to elaborate on decellularization techniques or use some additional post-decellularization procedures. Herein, we discuss apoptosis-assisted decellularization, as well as efficient antigen removal methods, crosslinking, and sterilization as the main modifications to address the immunogenicity issue in the dECM scaffolds.

### Apoptosis-assisted decellularization

Apoptosis induction is a novel approach for obtaining acellular scaffolds with favorable immunogenicity. In common decellularization techniques, necrosis is the primary mechanism of cell death and accompanies vast leakage of intracellular immunogen ingredients. The ingredients reattach to ECM and enhance the immunogenicity of the created dECM scaffolds. Apoptosis-assisted decellularization is based on programmed cell death, fragmentation, and retention of cell remnants within the cell membrane [170]. Apoptosis-assisted decellularization eventually inhibits the massive release of ECM inherent cytokines, which is common in more aggressive methods [171]. Ideally, dsDNA breaks into 180 base pair length fragments during apoptosis, concordant with the suggested decellularization criteria [172]. Furthermore, apoptosis loosens cell membrane-ECM integrity and creates minute apoptotic particles (0.5-2 µm), which could get easily washed and removed out of ECM with minimal harm to its structure [173]. In addition to inhibiting the leakage of immunogen cellular constituents, phagocytosis of remaining apoptotic bodies after decellularization by macrophages would also alter cytokine production patterns. Inhibition of TNF-α secretion and increase in TGF-β1 and PGE2 release are the significant cytokine-related impacts, exerting an immunomodulatory effect [174, 175]. Likewise, DCs phagocytose apoptotic bodies, process and present them to T cells via distinct mechanisms, which mediate a less aggressive immune response. The findings are consistent with the significantly lower amount of danger signals, which have a critical role in T cell activation in the apoptotic context compared to necrosis [175].

**Fig. 3:**
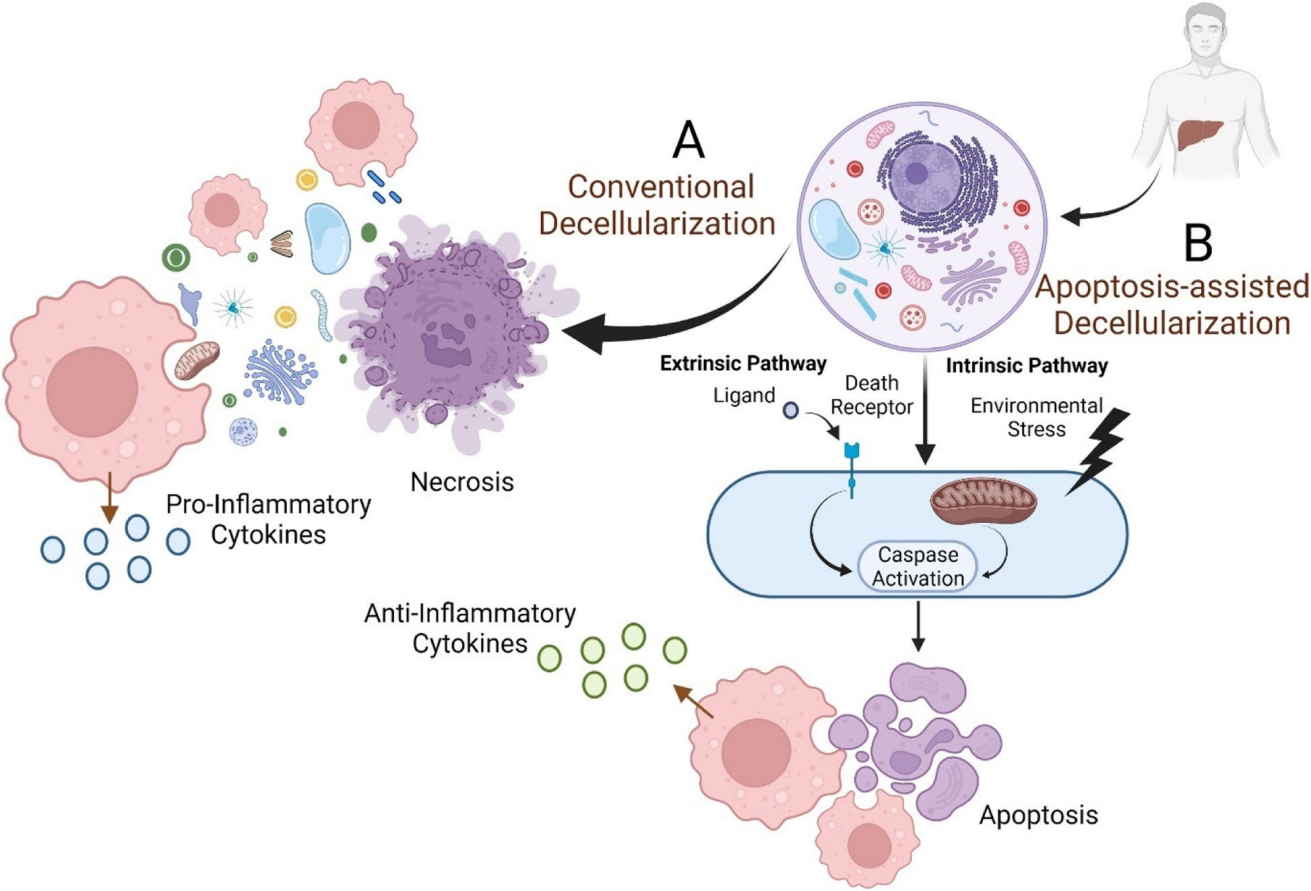
Apoptosis and necrosis as the core mechanisms of cell death in decellularization. Necrosis or apoptosis is the ultimate fate of cells upon the decellularization process. **A** Cell necrosis resulting from applying conventional decellularization techniques drives the vast leakage of immunogenic cellular materials, which invokes inflammatory responses following the implantation of derived scaffolds. **B** Apoptosis-assisted decellularization techniques exploit environmental stresses (extrinsic pathway) or death ligands to induce apoptosis. Following apoptosis, immunogenic cellular contents are degraded and confined to membranous vesicles that exert an immunomodulatory response

Extrinsic and intrinsic pathways are the principal mechanisms deployed for apoptosis induction. The extrinsic pathway relies on death ligands to stimulate death receptors and activate caspases, which lead to apoptosis (Fig. 3). Death receptors subtypes and their response to the ligands vary from tissue to tissue, necessitating tissue-specific adjustment. Complications with the entrapment of some ligands in ECM and their immunogenicity, besides high expenses, are considered this pathway’s main drawbacks [173]. The intrinsic pathway, also called the mitochondrial pathway, exploits environmental stresses to activate the apoptosis cascade. Affordability and simplicity of setup are the main superiorities, while the incomplete decellularization and their high reliance on elaborating various parameters such as oxygen content, temperature, pressure, and pH are the main hurdles to be taken.

As an example of apoptosis-assisted decellularization, N2-induced hypoxia can remove cell materials from porcine cornea [74]. Thermal changes, unlike the freeze and thaw method, which results in cell necrosis, need to be regulated within a range of temperatures between 10 °C and 45 °C to adopt apoptosis [173, 176]. Likewise, relatively lower pressures (around 200 MPa) in high hydrostatic pressure decellularization techniques inhibit excessive DAMP release via induction of shift in the cell death mechanism toward apoptosis in bone, cartilage, and dermal tissues [177, 178]. Both basic [179] and acidic environments could be delicately exploited to induce apoptosis-assisted decellularization [180]. Comelison et al. used camptothecin, an inhibitor of DNA topoisomerase-I, to decellularize rat peripheral nerve tissue via activation of the intrinsic pathway. They demonstrated self-sufficiency of apoptotic decellularization and lower immunogenicity of the derived scaffolds compared to detergent-based strategies. In their study, the substitution of hypertonic PBS for detergents resulted in the perfect preservation of ECM components. Another advantage of their apoptotic decellularization was the exclusion of initial tissue rinsing with distilled water, which is applied in many decellularization protocols to lyse cells at the cost of hurting ECM architecture and scattering antigens [181]. To achieve maximum efficiency, extrinsic and intrinsic pathways could be deployed together, combined with genetic engineering techniques, which promote the expression of death receptors on the cells [182, 183]. Apoptosis-assisted strategies are also compatible with perfusion bioreactors, enhancing their ability to decellularize different organs [171].

### Antigen removal

Decellularization protocols have distinctive abilities in removing each immunogenic cell protein. While a decellularization process successfully removes cytosolic immunogen proteins, it might be unable to remove immunogen cytoskeleton proteins effectively [46]. The heterogeneous results of some decellularization protocols in removing immunogenic factors make applying robust antigen removal steps necessary to overcome the immunogenicity issue [184]. Regarding their specificity in targeting immunogens, there are two main methods to efficiently reduce the immunogenicity of dECM, including selective antigen removal and solubilization.

#### Selective antigen removal

Known antigens, such as α-gal, could be exclusively eliminated via enzymatic treatment. α-galactosidase utilization is cost-effective in removing α-gal epitopes without ECM damage [130, 185]. Stone et al. utilized α-galactosidase treated porcine tendons to reconstruct injured anterior cruciate ligaments (ACL) of six patients. They observed the efficacy of enzymatic treatment in modulating the transplants’ immunogenicity. Successful conversion of xenografts to humanized ACLs occurred in five out of six patients over two years, and the resulting ACLs performed their optimal function through 10 years of follow-up [116]. Likewise, PNGase F enzymatic treatment has recently shown efficiency in removing glycan antigens (α-gal, Neu5Gc, and Sda). In a preclinical study, decellularized porcine heart valves, which were enzymatically treated with PNGase F, were less immune-reactive in sheep recipients. Accordingly, PNGase F could be utilized to ameliorate the immunogenicity of xenogeneic dECM scaffolds [49, 186].

#### Solubilization

Acellular scaffolds have a variety of known and unknown protein antigens with heterogeneous solubility, classified into hydrophilic and hydrophobic [187]. Sequential solubilization is an efficient antigen removal method that could be applied following the cell removal process to efficiently augment its efficacy in decreasing the antigenicity of derived dECM scaffolds. Sequential solubilization employs salts and reducing agents, and amidosulfobetaines to remove hydrophilic and hydrophobic, respectively [46, 187]. Depleting known and unknown antigens (e.g., minor histocompatibility antigens) significantly reduces the immunogenicity of dECM scaffolds. Unlike the level of DNA retention, the amount of remaining hydrophilic and hydrophobic antigens precisely correlates with the immunogenicity of the dECM scaffolds [25, 46]. In conventional decellularization methods, the oxidative environment precipitates proteins via thiol residue bounds and complicates their elimination. Maintenance of salts within an elaborated range has been shown to decrease protein aggregation. Therefore, salts such as KCl and reducing agents such as dithiothreitol are exploited to enhance the extraction of hydrophilic antigens [130]. The selection of appropriate salt is of the highest importance. Salts containing magnesium, calcium, and other divalent cations may precipitate nuclear antigens and hinder the solubilization and elimination of nuclear antigens from ECM. They also may co-precipitate DNA-related proteins [94].

Hydrophobic antigens, mainly from the membrane, seem to play a more critical role in the immunogenicity of dECM scaffolds. Sulfobetaines are a subgroup of zwitterionic detergents with good efficacy in solubilizing proteins and negligible compromise on ECM ultrastructure. They have relatively higher potency in extracting hydrophobic antigens than ionic detergents and seem to be the most effective agent in eliminating hydrophobic xenoantigens [88]. In a preclinical study, amidosulfobetaine-16 (3% w/v) successfully removed 92% of total hydrophobic antigens (99% reduction in both α-gal and MHC I) in the bovine pericardium. It produced bioprosthetic heart valves with the desired remodeling and comparable immunogenicity to glutaraldehyde (GA) crosslinked valves which have previously been shown to prevail over adaptive immunity [88]. Hydrophobic solubilization is also efficient in de-lipidation and impedes the calcification of dECM scaffolds via the depletion of negatively charged lipids [46].

### Crosslinking to reduce the immunogenicity of dECM

Crosslinking reduces decellularized tissues’ immunogenicity by restoring the structural distortions that occurred during decellularization. These distortions in collagen α-helix 3D structure may expose hidden antigenic sites and provoke immune reactions, leading to graft rejection [162]. Elastin denaturation during the decellularization may also produce elastin particles with significant antigenicity [115]. An optimized crosslinking strategy could add the advantages of retaining dECM properties similar to native tissue and decreasing its immunogenicity via masking the remaining antigens [188, 189]. Nevertheless, heavy crosslinking, especially with chemical agents, dampens dECM biodegradability and results in a higher M1/M2 ratio in macrophage polarization, more chronic inflammation, and FBR compared to non-crosslinked analogs following implantation [190]. Furthermore, some crosslinkers may denature collagen and expose its hidden antigenic sites or produce dECM scaffolds, which release cytotoxic and immunogenic by-products [191]. Distinctively, each crosslinking technique may also exert some adverse effects on ECM; for instance, water-insoluble crosslinkers require solvents, which may denature ECM proteins and reduce crosslinking efficacy, and such treated tissues usually provoke immune reactions upon implantation [192].

Over and above that, mechanical and topographic properties of decellularized tissues are critical players in the host immune response against implanted dECM scaffolds. Alteration in dECM natural conditions may affect macrophage polarization, degradation process, and FBR incidence [193]. The application of preparation techniques such as homogenization, which compromise the mechanical integrity of the dECM scaffold, may result in the excessive release of inherent cytokines and trigger more intense inflammatory responses [194]. Decellularization inevitably depletes GAGs and other ECM compositions and drives scaffolds with inferior mechanical strength. To address these challenges, crosslinking has proved its efficacy in promoting mechanical stability and inhibiting the rapid degradation of decellularized tissues, providing sufficient time for angiogenesis by endothelial cells and new ECM synthesis [195, 196]. Taken together, crosslinking is a double-edged sword, and accurate control of the degradation rate is required to achieve an equilibrium between graft stability and functional remodeling [190, 195]. Crosslinking strategies are classified into two principal categories: chemical and physical crosslinking.

#### Chemical crosslinking

GA is the most renowned chemical crosslinker, widely applied to crosslink bioprosthetic heart valves [197]. The popularity of this water-soluble crosslinker is attributed to relatively high crosslinking efficacy, wide availability, and immunomodulatory effects. GA crosslinks different protein chains via the interaction of aldehyde groups with amine groups of lysine or hydroxylysine. This crosslinker increases ECM resistance to chemical and enzymatic degradation [198, 199] and, most importantly, alleviates the immunogenicity of biological implants and consequently enhances their longevity via masking immunogenic sites [198]. Despite the efficacy of GA treatment in decreasing hyperacute and acute rejection of xenogeneic tissues, GA crosslinking does not fully inactivate antigenic sites, and grafts would still be susceptible to delayed immune recognition and chronic rejection [117, 200]. Undesirably, GA application per se confronts significant hurdles associated with its immunogenicity, cytotoxicity, and calcifying effects. Long-standing leach of GA residues and free aldehyde groups out of GA-treated scaffold prolongs these detrimental effects [197, 199, 201]. Moreover, in typical concentrations, its high crosslinking density may interfere with tissue regeneration by inhibiting the biodegradation of scaffolds even up to two years and is associated with a higher M1/M2 ratio, chronic inflammatory response, and FBR [190, 198]. To minimize these adverse effects, dECM could be treated with low concentrations of GA [199, 202]. Likewise, neutralizing agents such as glycine, hyaluronic acid, heparin, and chitosan have shown promising results in detoxifying free aldehydes and reducing immunogenicity and calcification in GA-treated dECM scaffolds [198, 202]. In the previously discussed clinical trial, α-gal depleted porcine tendons, which were partially crosslinked with a low concentration of GA (0.1%) and detoxified with glycine solution, were successfully remodeled in patients. The porcine tendons exhibited an immune response equilibrium, allowing fibroblast alignment [116]. However, GA prohibits the release of substantial factors for tissue differentiation, which might compromise its application in sites other than connective tissue [47]. To overcome cytotoxicity and other GA-related deficiencies, various alternatives have been evaluated, including natural crosslinkers such as chondroitin sulfate [162], genipin and quercetin [203], procyanidin [204], enzymatic crosslinkers such as transglutaminase,[199] carbodiimides such as EDC [189], isocyanates, carbohydrates such as ribose and glucose, photoreactive agents such as riboflavin and rose bengal [190] and poly epoxy compounds [205].

Natural crosslinkers such as chondroitin sulfate are frequently used biocompatible agents. Crosslinking with chondroitin sulfate simultaneously ameliorates the mechanical properties of dECM scaffolds and decreases inflammatory response by impeding the NF-κB transcriptional pathway in the immune system. In a preclinical study, the application of chondroitin sulfate for crosslinking acellular goat corneas significantly reduced their immunogenicity following implantation into rabbits and promoted host regenerative processes [162]. Genipin is another natural crosslinker with good biocompatibility, regenerative features, and immunomodulatory effects on the dECM scaffolds [191, 201, 203]. Genipin reduces the antigenicity of decellularized tissues by decreasing free amino groups with the same mechanism as GA to maintain structural integrity [195, 206]. Comparative preclinical studies on porcine decellularized esophagus and liver demonstrated that despite GA-processed dECM, genipin-treated scaffolds induced a shift in infiltrating macrophages toward the M2 subtype and decreased the number of infiltrating pan-macrophages upon their implantation into rats [207, 208]. Genipin also alleviates immunogenicity by impeding CD4^+^ T cell proliferation and pro-inflammatory cytokine secretion [191] and has beneficial effects on the angiogenesis of implanted dECM scaffolds [203, 208]. In spite of being much more biocompatible than GA, several studies have reported cytotoxic effects for genipin. Its cytotoxicity is dose-dependent but time-independent, and in concentrations up to 0.5 mM has optimal biocompatibility and is considered safe in most tissues [199, 201]. Procyanidin is the other naturally derived crosslinker with favorable biocompatibility. Crosslinking decellularized rabbit uterus with procyanidin resembled genipin treatment in terms of immunogenicity following implantation in rat recipients [192]. Wang et al. crosslinked pure aortic elastin with procyanidin and demonstrated its efficacy in blocking calcification nidi, decreasing macrophage-ECM interaction, and prohibiting their pro-inflammatory cytokines and MMPs release. Procyanidin enhances elastin resistance to MMPs mediated degradation, and its application lowers the immunogenicity and calcification of treated dECM [204].

1-ethyl-3-(3-dimethylaminopropyl) carbodiimide (EDC) is another chemical crosslinker that has recently gained interest in reducing the immunogenicity of dECM scaffolds [209]. Despite GA and genipin, EDC crosslinks proteins without being a part of the final scaffold but with less potency, making the cytocompatibility of crosslinked products negligible. EDC is often applied in combination with N-hydroxysuccinimide (NHS), which promotes EDC crosslinking potency and enhances final product stability. However, EDC/NHS crosslinking significantly blocks cell attachment sites, and its treatment concentration needs precise regulation [199]. EDC/NHS treated dECM scaffolds have shown remarkable mechanical similarity to native tissue and biocompatibility for mesenchymal stem cells in different tissues without induction of adverse immune response [189, 201, 210]. Both EDC and NHS are water-soluble, and the ease of their elimination via simple washing steps provides additional superiority for EDC/NHS treated scaffolds biocompatibility [201].

#### Physical crosslinking

UV irradiation and dehydrothermal treatment are common physical crosslinking methods that offer simultaneous sterilization with no need for cytotoxic chemicals [211, 212]. In dehydrothermal treatment, dECM scaffolds are heated under vacuum conditions. It is a safe and biocompatible technique that relies on water molecule depletion to exert dehydrothermal treatment crosslinking influence. It successfully reduces the immunogenicity of dECM scaffolds while increasing the mechanical integrity and pore sizes, making them suitable for subsequent cell attachment [213].

Nonetheless, UV-irradiated dECM scaffolds are inferior in biocompatibility because UV depletes growth factors and denatures proteins. UV also has a low penetration potency making it available only for thin dECM scaffolds. To address these limitations, UV is usually applied in combination with a photosensitizer [211]. Using acryl groups, namely Acrylation, is the most widely utilized method to induce photosensitization. However, after crosslinking, unreacted acryl groups provoke immune responses [214]. The application of riboflavin as a natural vitamin is a promising photosensitizer in terms of biocompatibility. In a preclinical study, subcutaneous implantation of riboflavin-UV-treated decellularized porcine heart valves in rats demonstrated a significant reduction in the number of inflammatory cells compared to untreated dECM scaffolds [215].

### Sterilization to eliminate pathogen-related immunogenicity of dECM

Similar to other medical devices, dECM scaffolds can get contaminated by pathogens and endotoxins, which enhances their immunogenicity in a tissue- and dose-dependent manner. On the one hand, FDA regulatory criteria have put a maximum 0.5 EU/ml threshold of endotoxin contamination for medical devices, including dECM scaffolds [75]. On the other hand, implantable medical devices are not allowed to have pathogen contamination with a probability of higher than one in a million [216]. Accordingly, the utilization of suitable sterilization techniques in manufacturing dECM scaffolds is of the highest importance to avoid infection and rejection [217]. Although various sterilization techniques have been applied for this purpose, many of them utilize aggressive mechanisms which may exert detrimental effects on the structure and immunogenicity of ECM [218]. Some sterilization methods may increase FBR incidence via crosslinking ECM components and interfering with subsequent degradation [219]. Among various agents and techniques, ethanol, antibiotics, PAA, ionizing radiations, ethylene oxide, electrolyzed water, and supercritical CO2 are the most commonly applied sterilizers for dECM scaffolds.

Ethanol 70% denatures polypeptides and is incapable of bacterial spore elimination, which makes it an inferior choice for sterilizing dECM scaffolds [218]. Antibiotics and antimycotics like penicillin, streptomycin, and amphotericin B are common disinfectants with favorable ECM preservation. Nonetheless, incomplete coverage of pathogens has restricted their use to experimental research [69] or made them an adjuvant for other sterilization methods such as PAA treatment [220]. Besides the ability to remove cellular materials, PAA has also shown promising results in terms of dECM sterilization. Concomitant DNA removal and desirable ECM preservation reduce the PAA-treated scaffold immunogenicity. Although ECM might be subject to some negligible deleterious effects due to PAA acidity, the cytocompatibility of PAA makes it a remarkable option for the sterilization of dECM scaffolds [220, 221].

Ionizing radiations, including γ-irradiation and electron beam irradiation, are prominent physical sterilizers, inducing nucleic acid denaturation to terminate pathogen activities. They may damage ECM ultrastructure and produce lipid-derived cytotoxic particles that have limited their application. Mild irradiation induces crosslinking of dECM scaffolds, while heavy irradiation denatures and degrades ECM, resulting in inferior mechanical strength and cell attachment capability. In this regard, 25 kGy is the optimal Gama dose for sterilization of dECM scaffolds with favorable ECM preservation and induction of minimal immune response.[56, 222].

Although ethylene oxide sterilization is a very effective and widely applied sterilization method, it is toxic and carcinogenic and is demonstrated to release immunogen residues and dampen scaffold biocompatibility [223]. Of note, Gamma irradiation and Ethylene oxide sterilization also alter collagen 3D ultrastructure and deform ECM binding sites for cell alignment, which results in inferior biocompatibility of ECM-based biomaterials [218].

**Fig. 4:**
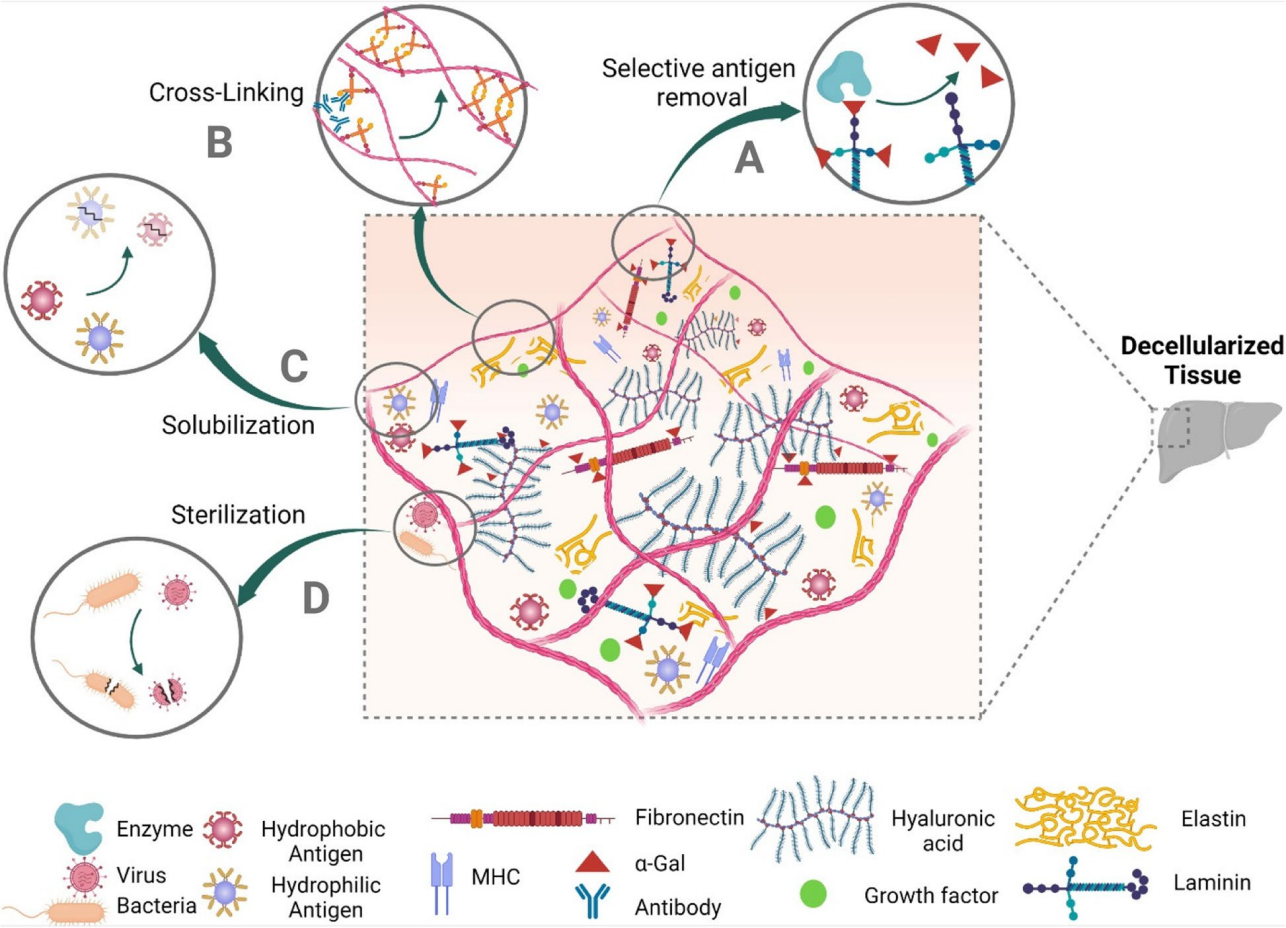
Common post-decellularization modifications to address the immunogenicity of dECM scaffolds. **A** Selective antigen removal techniques rely on enzymatic treatment to specifically remove antigens (i.e., α-gal, Neu5Gc, and Sda epitopes). **B** Crosslinking may reduce the immunogenicity of dECM by masking exposed antigenic sites. **C** Solubilization removes various protein antigens (hydrophobic and hydrophilic antigens) according to their common physiochemical properties. **D** Terminal sterilization eliminates pathogen-related immunogenicity of viruses and bacteria in the dECM scaffolds

Recently, some researchers have proposed the application of mild acidic electrolyzed water and supercritical CO2 as novel techniques to sterilize decellularized tissues with minimal adverse effects on ECM ultrastructure. Due to some encouraging data related to their efficacy in removing viruses and bacterial spores, the impact of these sterilization techniques on the immunogenicity of dECM scaffolds is worth further investigation [78, 221]. Depending on each tissue’s unique feature, the best sterilization methods are yet to be tailored to the tissue of interest. Figure 4 shows the main post-decellularization modifications to ameliorate the immunogenicity of dECM scaffolds.

## Conclusions and future perspective

Although recent advances have paved the road for the clinical application of scaffold-based engineered tissues/organs, some immunological issues remain critical concerns and areas for future research. Challenges with the immunogenicity of dECM scaffolds are categorized under several headings, including (i) decellularization protocols should be adjusted to efficiently remove cellular materials and better preserve ECM and maintain its integrity; (ii) effective antigen removal techniques are required to eliminate remaining antigens post-decellularization; (iii) practical detection measures and more efficient washing techniques should be developed to eliminate residues of cytotoxic agents applied in some decellularization protocols; (iv) tissues/organs should be obtained from more immunocompatible sources; (v) degradation rate of dECM scaffolds should be adjusted to avoid either FBR and chronic immune response or accelerated release of inherent cytokines and graft failure; (vi) suitable sterilization method should be tailored to minimize ECM damage and immunogenicity in a tissue/organ-specific manner; (vii) a standard for acceptable immunogenicity should be established enabling us to predict the in vivo outcomes and better decide which dECM scaffold meets the prerequisites for clinical translation. Addressing these challenges will catalyze substituting conventional transplants with widely available personalized tissue-engineered analogs. For this purpose, practical approaches are under investigation with a focus on improving the recellularization process and providing decellularization sources with less immunogenicity.

Fine-tuning the degradation rate of dECM scaffolds is a critical approach to ameliorate their immunogenicity via optimizing the subsequent recellularization and regeneration process [224]. Crosslinking is a long-established method to slow down the degradation process and impede rapid graft degeneration. However, accelerating the degradation rate might be necessary to avoid unfavorable immune reactions. Some novel strategies, including growth factor, exosome, and α-gal nanoparticle therapy, could be exploited for this aim. One strategy relies on growth factor therapy in order to facilitate the host cell recruitment and regeneration process. Local administration of growth factors is usually difficult, and their systemic administration is inefficient due to poor in vivo stability and undesired side effects. Systemic administration of nanocapsules formed by polymerization of growth factors and MMP-cleavable peptides could solve these problems. After dECM implantation, MMPs are released by invading macrophages and other recipient cells and provide localized nanocapsule-mediated growth factor delivery and improve the recellularization process [225]. Moreover, mesenchymal stem cell-derived exosomes enriched with growth factors and anti-inflammatory cytokines could be administrated to accelerate the regeneration process as well as subsiding immune reactions [226, 227]. Another approach is α-gal nanoparticle administration to induce rapid antigen-antibody interaction and, consequently, macrophage migration and chemokines secretion. Thereby, progenitor/stem cell recruitment and degradation occur faster. This process does not adversely affect the ECM in the absence of α-gal epitopes within the graft. It might also provide significant superiority in avoiding non-gal-related humoral immunity [228]. Following implantation of xenografts, α-gal antibodies peak within two weeks, while anti-non-gal antibodies require one [47] to six months [116]. This difference is presumably because anti-non-gal antibodies are produced by various B cell clones, each initially having a few activated cells. This delay in antibody production suggests accelerated progenitor/stem cell recruitment as a potential approach to negate the detrimental effects of anti-non-gal antibodies attachment to ECM [47].

Considering the wide variety of immunologic challenges associated with xenogeneic dECM, providing decellularization sources with less immunogenicity is another important issue. One approach is to exploit novel bioengineering methods, such as nuclease-based genome editing, which have paved the road for xenotransplantation (vital organs). Exposing cells of GT-CMAH knockout pigs (which lack expression of α-gal and Neu5Gc epitopes) to human serum demonstrates fewer antigen-antibody reactions [133, 134]. Harvesting tissues or organs from genetically manipulated pigs with suppressed α-gal, MHC I, and MHC II productions, simultaneously reduces antibody and T cell-mediated immunogenicity and may enhance xenograft longevity [229]. Although the immunogenicity of these xenografts does not meet the requirements for clinical translation yet, organs derived from such animals could supply decellularization material and attenuate the immunogenicity of derived scaffolds. A comparative study on the implantation of decellularized WT and GT knockout (α-gal free phenotype) porcine lungs into non-human primates demonstrated the superiority of GT knockout animal-derived scaffolds in terms of long-term immunogenicity and chronic T cell response [146]. However, regulatory issues, complexity, time-consuming process, and high costs are among the main barriers that need to be addressed in these methods [133, 185]. Another approach is the decellularization of alternative human organs with similar structural/chemical components as a suitable substitution for target organs. In one successful example, splenic dECM scaffolds have shown promising results for bioengineering the liver and pancreas. However, further studies should be done on this idea before translation into the clinic [230, 231].

In conclusion, despite encouraging results regarding the application of some dECM scaffolds, their immunogenicity has remained a challenging barrier that needs to be addressed. Recognition of contributing factors in the immunogenicity of dECM will enable us to find solutions to fabricate bioscaffolds with favorable biocompatibility, which could be ideal surrogates for conventional transplants in the near future.

## Data Availability

Not applicable.

## Abbreviations

ECM: Extracellular matrix
DAMPs: Damage-associated molecular patterns
MHC: Major histocompatibility complex
APC: Antigen-presenting cell
DC: Dendritic cell
FBR: Foreign body response
dECM: Decellularized ECM
ROS: Reactive oxygen species
PRRs: Pattern recognition receptors
IL: Interleukin
TNF: Tumor necrosis factor
MMPs: Matrix metalloproteinases
GAGs: Glycosaminoglycans
PAA: Peracetic acid
TX100: Triton X 100
SDS: Sodium dodecyl sulfate
SDC: Sodium deoxycholate
EDTA: Ethylenediaminetetraacetic acid
HMWHA: High molecular weight hyaluronic acid
TLR: Toll-like receptor
LMWHA: Low molecular weight hyaluronic acid
LAIR-1: Leukocyte-associated immunoglobulin-like receptors-1
A-gal: A galactose-alpha-1,3-galactose
Neu5Gc: N-glycolylneuraminic acid
SIS: Small intestinal submucosa
GA: Glutaraldehyde
EDC: 1-ethyl-3-(3-dimethylaminopropyl) carbodiimide
NHS: N-hydroxysuccinimide
